# Health literacy of people with spinal cord injury: a systematic review

**DOI:** 10.1038/s41393-023-00903-4

**Published:** 2023-06-30

**Authors:** Francine A. R. Silva, Maria A. Barbosa, Cejane O. M. Prudente, Letícia A. Morais, Katarinne L. Moraes, Vanessa S. C. Vila, Celmo C. Porto

**Affiliations:** 1 Health Sciences Program, Federal University of Goiás, Goiânia, Brazil; 2 School of Social and Health Sciences, Pontifical Catholic University of Goiás, Goiânia, Brazil; 3 Science and Technology in Health Program, University of Brasília, Brasília, Brazil; 4 Faculty of Ceilândia, University of Brasília, Brasília, Brazil

**Keywords:** Patient education, Spinal cord diseases

## Abstract

**Study design:**

Systematic review.

**Objective:**

To systematically review the evidence on health literacy (HL) of people diagnosed with spinal cord injury (SCI).

**Methods:**

PubMed, Cochrane Library, Web of Science and Embase databases were used to identify studies published from 1974 to 2021. Two reviewers independently carried out the study selection process and assessed the methodological quality of the studies. The risk of bias in the studies was classified according to the Joanna Briggs Institute (JBI).

**Results:**

In total, 1398 studies were identified from the initial search, and 11 were selected for reading thoroughly. After screening, five studies were included. All had a cross-sectional design, and most scientific production was from the United States. In the studies, people with SCI received assistance in rehabilitation services. The results were heterogeneous compared to the HL: reasonable HL; suitable HL; Inadequate HL. Better HL was identified in individuals from the white population compared to the black population with SCI.

**Conclusion:**

Studies on HL in the SCI population are limited. Guidance and personalized education provided in rehabilitation programs seem to have an influence on HL levels in this population. More research is needed to broaden the understanding of HL in the rehabilitation process of people diagnosed with SCI.

## Introduction

Spinal cord injury (SCI) can be defined as an injury of the spinal cord which can result in damage related to motor, sensory, and autonomic functions [1, 2], leading to physical, emotional, and economic consequences for patients, families and society in general [3].

People with SCI are prone to an increase in the number of comorbidities and a decrease in their physical abilities. Impairments resulting from this injury vary from one individual to another, which predisposes the person to a condition of functional incapacity, and the consequent need for continuous guidance [4, 5].

In this context, it is important to analyze how people with SCI understand and use the guidance provided by health teams to make decisions and act on self-care [6, 7]. Furthermore, these aspects are directly related to health literacy (HL) [8].

The World Health Organization defines HL as the “cognitive and social skills that determine the motivation and ability of individuals to gain access, understand and use information in ways that promote and maintain good health” [9].

Adequate HL becomes an empowerment tool for individuals by enabling access to and use of health information efficiently [6, 10]. Relevance of the HL assessment is evident among the population with SCI, mainly due to the necessary care, such as medical follow-up, pharmacological and non-pharmacological prescriptions [11].

This is a growing research area, mainly because inadequate HL is associated with worse physical and mental health, functional limitations, risks associated with morbidities and higher health care expenses [12–14].

This topic is still not fully addressed in rehabilitation research, or in the clinical practice of individuals with SCI. To the best of our knowledge, there are no systematic reviews in the literature that identify HL in patients with SCI.

Furthermore, the opportunity to assess the quality of primary studies published on HL in SCI should be mentioned. Thus, the objective was to systematically review the evidence on health literacy (HL) of people diagnosed with spinal cord injury (SCI).

## Methods

### Literature search strategy

The systematic review was conducted following the PRISMA (Preferred Reporting Items for Systematic Reviews and Meta-Analysis) recommendations [15]. The protocol of this review was registered in the International Prospective Register of Systematic Reviews (PROSPERO) database, under registration number CRD42020157252.

A comprehensive search strategy was developed, including the Medical Subject Headings (MeSH) terms, and an exploratory investigation was conducted to identify keywords referred to in articles in the area. The descriptors were combined as follows: health literacy OR literacy OR patient education as topic OR health education AND spinal cord OR spinal cord injury OR spinal cord injuries OR tetraplegia OR paraplegia. Details of the search strategy are provided in Table 1 of the Supplementary Material.

The electronic search was carried out in four databases PubMed, Cochrane Library, Web of Science and Embase, via the Capes periodical portal, from September to December 2021, aiming to answer the following guiding question: What is the health literacy of people like with Spinal Cord Injury?

### Inclusion and exclusion criteria

Studies were included adopting the following criteria: (1) adult individuals diagnosed with SCI, traumatic or non-traumatic types, of any gender, with paraplegia or tetraplegia, complete or incomplete types, from the general community, or health program participants, (2) studies that involved HL assessment, using validated instruments, from 1974 (the term Health Literacy that originated in English and was used for the first time by Scott Simonds in 1974) [7] to December 2021.

Editorials, letters, comments, reviews, case reports, dissertations, course final papers, qualitative studies, congress abstracts, and pre-prints (without peer review) were excluded.

### Selection process

Two reviewers (FARS and COMP) independently conducted the study selection process and the assessment of the methodological quality of the studies. After removing duplicate results, articles were selected by reading titles and abstracts, and the full text was used for rescreening. When considered eligible consensually by the two researchers, the articles were included in the study, discrepancies were resolved by consensus and, if necessary, a third reviewer was consulted (CCP).

### Data extraction

Data from selected studies were extracted and organized into tables, including the main author, title, year of publication, location of data collection, language, journal, objectives, study design, sample characteristics (sample size, gender, color, marital status, education, time of injury), instruments used in the HL assessment, the main results and associations with HL. Data extraction was performed by one of the authors (FARS).

### Risk of bias assessment

To assess the methodological quality of the studies, standardized critical assessment instruments were used, developed by the Joanna Briggs Institute (JBI) [16].

Two reviewers independently assessed the studies considered eligible, using the JBI Critical Appraisal Checklist according to the study design, and discussed the analysis, until reaching common ground. Moreover, two other researchers (CCP and MAB) participated to establish consensus in case of any questions raised during the assessment process.

The standardized form to assess the methodological quality of the studies consists of a checklist that contains items evaluated as: Y (yes), N (no), U (unclear) and NA (not applicable). If the primary study met all the criteria described, the answer “Yes” to the question was ticked. If the study did not develop/assess in the way described in the question or did not mention the item in question, the answer “No” was given. For any item, if it was not clear how that topic was developed, the answer “Unclear” was marked. Finally, if the question did not apply to what was being analyzed, the answer was “Not applicable” [16–18].

The risk of bias was classified according to the study by Almeida et al. [19], as high risk of bias (up to 49% of “yes” answers), moderate (50–69% of “yes” answers) and low (70% or more of “yes” answers).

## Results

In the present research, 1398 studies were identified. Of these, 1006 titles and abstracts were selected, and 11 studies were selected to read thoroughly. Five of these met the inclusion criteria. The selection is shown in detail in the PRISMA diagram [20] (Supplementary Fig. 1).

### Study location and design

Of the five studies selected for the final sample, four were from the United States of America (USA) [21–25], and one from Turkey [24].

The sample consisted of observational articles [21–25] with a cross-sectional design that assessed the HL of people with SCI, using five different psychometric instruments. The studies were published in various journals in English from 2005 to 2021. Table 1 provides an overview of the results of the five selected articles.

**Table 1:** Summary of the results of studies on HL in people with SCI.

Author year	Location language	Objectives	Sample profile	Instruments, results and associations related to HL
Hahn et al. [22] Health and Functional Literacy in Physical Rehabilitation Patients -2017	- Two academic medical centers and a rehabilitation hospital in the Midwest of the United States (Rehabilitation Institute of Chicago, University of Michigan Washington University in St. Louis) -English	- To describe the levels of HL in people with stroke, SCI and Traumatic Brain Injury (TBI) -To evaluate associations between HL, cognitive function and education, mobility, anxiety, and health status.	-209 individuals with traumatic SCI, most male (78%), mean age 43 years, white (60%), married (36%), about 1/3 of each group had high school or higher education, mean time of injury of 12 years.	- The group with SCI had higher HL levels (Health LiTT = 58.1) compared to patients with CVA (Health LiTT = 53.6) and TBI (Health LiTT = 57.8) -Adequate HL was observed, and it was correlated with better cognition, mobility, less anxiety and better self-reports of general health.
Hogan et al. [21] Health Information Seeking and Technology Use Among Veterans with Spinal Cord Injuries and Disorders - 2016	-Veterans Rehabilitation Center (VA Medical Centers), located in Washington -English	- To characterize the search for health information among veterans with SCI. -To examine association between use of technology, self-reported health status and HL in veterans with SCI.	-290 people with SCI, mostly male (97.2%), under 65 years of age (71.0%), white (71.7%), married (58.6%), injury time of less than 10 years.	- Overall average eHEALS score was 27.3, which corresponded to reasonable levels of HL. -Veterans who reported good or excellent health status scored higher on the HL assessment. -There were no associations of HL with the use of technologies.
Johnston et al. [25] Health Literacy, Morbidity, and Quality of Life Among Individuals with Spinal Cord Injury -2005	-Kessler Institute for Rehabilitation, New Jersey -English	- To describe the levels of HL in health in the SCI and investigate their possible associations with educational levels, morbidity, QoL, functional independence, community participation and satisfaction with life.	-107 participants with SCI, male predominance (82.2%), mean age of 39 years, white (66%), single (65.4%), higher education (29%), mean time of injury of 8.7 years.	- HL adequate in TOFHLA (86%). -Better HL result was related to better education, lower physical morbidity, and better life satisfaction.
Myaskovsky et al. [23] The association of Race, Cultural Factors, and Health-Related Quality of Life in Persons with Spinal Cord Injury -2011	-Six specialized rehabilitation centers in SCI, the United States of America (USA) -English	-To examine the association between HL, racial and cultural factors, QoL, social participation, life satisfaction, perception of health status in people with SCI.	-252 people with SCI, most of them male (76.2%), under 50 years of age, 156 white and 96 black, most of them married (35.7%), higher education (36.9%), mean injury time of 12 years	- REALM showed a lower HL among African Americans (6.95 ± 1.47) compared to whites (7.63 ± 1.18), and an association between HL and race can be observed. -African Americans also reported more experiences of discrimination in health care, greater perceived racism, and distrust of the health care system.
Sertkaya, et al. [24] Investigation of health literacy level and its effect on quality of life in patients with spinal cord injury -2021	-Research Hospital, Education in Physical Medicine, and Rehabilitation of Turkey -English	-To determine the level of HL in patients with SCI and its relationship with QoL.	-77 individuals with SCI, 53 males (68.8%), mean age of 37.3 years, married (62.3%), with length of study in years, between zero and eight (59.7%), and duration of injury less or equal to 1 year.	-The HLS-TR evaluated the level of HL as inadequate in 32.5%, problematic-limited in 40.3%, sufficient in 19.5% and excellent in 7.8% of patients. -Better results for the domains vitality and mental health in the QoL assessment were associated with better levels of HL.

*HL* health literacy, *CVA* cerebrovascular accident, *SCI* spinal cord injury, *TBI* traumatic brain injury, *USA* United States of America, *Health LiTT* Health Literacy Assessment Using Talking Touchscreen Technology, *eHEALS* e-Health Literacy Scale, *TOFHLA* Test of Functional Health Literacy in Adults¸ *REALM* Rapid Estimate of Adult Literacy in Medicine, *QoL* quality of life, *HLS-TR* The European Health Literacy Questionnaire Turkish Adaptation.

### Sample characteristics in the studies

The number of people with SCI evaluated in the studies ranged from 77 to 290. The study with the largest sample was carried out at the Veterans Health Administration (VA Medical Centers) [21], located in Washington (United States of America).

People diagnosed with SCI were recruited mainly from medical centers that served this profile of individuals [22], rehabilitation institutes [25], rehabilitation centers [21, 23], and rehabilitation hospitals [24] and presented injury time that varied from less than or equal to 1 to 12 years.

A study was carried out with individuals diagnosed with paraplegia and complete tetraplegia [21], but they did not specify the number of participants in each group. In three studies, most patients had paraplegia [21–24], and in another, tetraplegia [25]. The etiologies of SCI were not described in detail in the selected articles.

Most of the population was male, married, with varied education, predominantly white race/ethnicity, and in one of the studies this last characteristic was not mentioned [24].

### Instruments and procedures used in the Health Literacy assessment

Five different instruments were identified that mainly assessed the functional level of the HL, except for one of the instruments, where the main focus was digital HL. Table 2 summarizes the instruments used in the HL assessment including the descriptions made in the articles.

**Table 2:** Summaries of the descriptions of the HL assessment instruments in the studies.

Study	Instrument	Summary of the instrument
Hahn et al. [22]	Health LiTT	It is a self-administered multimedia test. Participants answer three types of items: prose (reading comprehension), document (identifying and interpreting information presented in charts, graphs, or tables) and quantitative (performing arithmetic operations). Each item has a multiple-choice answer format and only one answer is coded as correct.
Hogan et al. [21]	eHEALS	A scale used to measure the level of digital health literacy. It consists of eight items aiming to measure the knowledge, comfort and perceived skills of individuals on how to find, evaluate and apply electronic health information to health problems.
Johnston et al. [25]	TOFHLA	It has 50 items that test the ability to read and understand medical information, it presents 17 items related to the investigation of numeration, which consists of assessing the ability of using numbers in everyday life.
Myaskovsky et al. [23]	REALM	It comprises eight items. While applying it, the participants must read aloud a list of medical words. A correct answer is given if the word is read correctly.
Sertkaya et al. [24]	HLS-TR	It has 47 items and can also be used to measure HL sub-dimensions related to treatment services, disease prevention and health improvement.

*HL* health literacy, *Health LiTT* Health Literacy Assessment Using Talking Touchscreen Technology, *eHEALS* e-Health Literacy Scale, *TOFHLA* Test of Functional Health Literacy in Adults, *REALM* Rapid Estimate of Adult Literacy in Medicine, *HLS-TR* The European Health Literacy Questionnaire Turkish Adaptation.

In one study, the eHEALS, a scale developed to measure digital health literacy, and a cover letter explaining the nature of the study were sent to participants through the mail [21]. The other studies applied their instruments directly with the participants in the research fields, through interviews [23–25]. Furthermore, Sertkaya et al. [24] cited the self-reported measurement of HL.

Johnston et al. [25] used the TOFHLA instrument and mentioned that, although its reliability and validity are reasonably well established, this test had not been previously used in HL assessment in people diagnosed with SCI.

### Evidence on health literacy and associated factors

The articles included in this review obtained the following results concerning HL: reasonable HL [21]; adequate HL [22, 25]; Inadequate and problematic/limited HL [24]. Better HL in individuals from the white population compared to the black population with SCI [23].

The study that assessed digital health literacy in veterans with SCI [21] found an overall average score on the eHEALS scale of 27.3, which corresponded to reasonable levels of self-perceived literacy.

Adequate levels of HL were identified in most people with SCI treated at a private rehabilitation clinic [25]. The same result was found in the study by Hah et al. [22] who also identified better results for HL in people with SCI compared to individuals with Stroke and Traumatic Brain Injury.

The study carried out at the Hospital for Research, Education in Physical Medicine and Rehabilitation in Turkey evaluated the predominance of inadequate and problematic/limited HL in 72.8% of the population with traumatic SCI [24].

African Americans reported experiences of discrimination in health care, higher perceptions of racism, more distrust of the health care system, and lower HL than whites [23].

Among the studies, it was observed that adequate HL was associated with greater mobility, better cognition and less anxiety [22], as well as higher levels of education, less physical morbidity and better satisfaction with life [25]. In two studies, reports of better health status were related to reasonable [21] and adequate levels of HL [22].

### Quality of evidence

The included studies had a cross-sectional design. The JBI Critical Appraisal Checklist for Analytical Cross-Sectional Studies [18] was used. This checklist consists of eight questions and is applied in the critical evaluation of studies.

In the risk of bias analysis, studies were classified as having low risk of bias. In one of the articles, the main sources of bias were limitations in detailing the setting and study subjects, not clearly describing the participant selection process, including sociodemographic data, location and period [22].

In another study, when measuring the results item, training researchers to use the applied evaluation instruments was not described [25] and, finally, in the third study, the inclusion criteria developed before recruiting study participants were not clearly defined [24].

Two studies [21, 23] obtained better evaluations, reaching the maximum score in the Critical Appraisal Checklist for Analytical Cross-Sectional Studies. Full details of the risk of bias assessment are provided in Table 2 of the Supplementary Material.

## Discussion

This systematic review identified and summarized the results of studies that evaluated HL in adult individuals with SCI complications. Knowledge about HL in patients with SCI is very important information [26], however, it has not been fully addressed in the literature [22].

We observed adequate HL in two [22, 25] out of the five studies. In the others, we found variability in the results related to the level of HL in the population with SCI. One of the studies evaluated reasonable HL [21], in another inadequate and problematic/limited HL [24], and better HL was identified in individuals from the white population, compared to the black population with SCI [23].

In all included articles, researchers evaluated the HL of people with SCI who attended rehabilitation services [21–25]. The educational programs carried out in these centers aim to teach people to understand the necessary skills to carry out their activities of daily living, which consequently can influence HL levels [22, 27]. Confirming these findings, Floríndez et al. [28] observed circumstances that led to the development of pressure injuries in adults with SCI and concluded that professionals need to identify the HL to provide appropriate and understandable education.

Individuals with SCI, stroke and TBI in rehabilitation for ~1 year had adequate HL [22]. In the same study, the average time of injury varied between participants: 12 years for people with SCI, 3 years for stroke and 6 years for the group of people with TBI. Individuals in rehabilitation processes, with longer duration of injury, have appropriate management and perception of the disease, which may result in better HL levels [21].

Hahn et al. [22] also identified higher scores in HL assessment and cognition in people with SCI, when compared to groups with stroke and TBI. These results are similar to those reported in three other articles that identified a positive relationship between HL level and cognitive function [6, 12, 14].

The educational level of individuals with SCI and the characteristics of the rehabilitation services they attend may interfere with the HL results. Johnston et al. [25] identified limitations in the HL in a minority of people with SCI. The authors mentioned that this study was carried out in a private rehabilitation clinic, where most of the participants had a higher education qualification, which could explain the evaluated HL. Another study evaluated the HL through an open-ended question about the confidence of a person with SCI in obtaining the necessary information to minimize complications and found better HL in those with a higher level of education [4].

One of the studies [21] assessed the level of digital HL through eHEALS and health information sources used by veterans with SCI. The overall average score corresponded to reasonable levels of self-perceived digital HL. According to the authors, veterans with SCI reported that health professionals were the main sources used to obtain information about SCI, as they had more contact with them, compared to other means of information. Studies have identified that HL levels are favored by interaction with professionals and may contribute to a better understanding of health information [12, 26, 27].

Ethnicity/race, education, and HL have been associated with health disparities in people with SCI [29]. African Americans with SCI described discrimination in health care, higher perception of racism and lower HL compared to white people [23]. These findings suggest that healthcare is experienced differently by people of different races who have SCI, and that healthcare professionals need to consider these factors when interacting with these individuals.

Epidemiological studies are important to determine regional data, as HL levels can vary between countries and regions. Among the included studies, only one of them was not carried out in the United States and identified inadequate HL in people with SCI treated at a Teaching and Rehabilitation hospital in Turkey. Another study, also carried out in Turkey, evaluated 4924 participants from the general community, and found inadequate or limited HL in 64.6% of the participants [30], therefore, the level of HL detected in people with SCI was similar to the HL level in the general Turkish population.

People affected by some pathology who have inadequate levels of HL often demonstrate little knowledge about the signs and symptoms of the disease [14], which can compromise the quality of life (QoL). Two studies did not find a significant correlation between the level of HL and QoL in people with SCI [24, 25]. One of the studies found a weak correlation between HL and QoL [23], and another observed an association only between the domains vitality and mental health of the Short form-36 instrument and adequate levels of HL [24].

### Strengths and limitations of the study

The strengths of this review include a search in four highly important databases, conducting the assessment of each article, the search process and selection of articles by two independent researchers, the risk of bias assessed through a widely used and reliable tool: Joanna Briggs Institute (JBI), and the use of PRISMA guidelines and the checklist to conduct the review.

All studies were cross-sectional and had heterogeneity of instruments used in the HL assessment. In addition, authors of included studies were not contacted for additional information on unpublished or planned studies.

## Conclusion

Studies on health literacy in the spinal cord injury population are limited. Our study provides a summary of the evidence of health literacy in people with spinal cord injury. The results were heterogeneous compared to health literacy and the following aspects were found: reasonable health literacy; adequate health literacy; inadequate health literacy. Better health literacy was identified in individuals from the white population compared to the black population with spinal cord injury.

Treatments in rehabilitation services, duration of injury, cognition, educational level, specific characteristics of rehabilitation services, frequent contact with health professionals, race/ethnicity and geographic aspects were pointed out in studies as possible influencers on health literacy.

People with spinal cord injury attended rehabilitation services, therefore guidance and personalized education seem to influence health literacy levels, however more research is needed to broaden the understanding of health literacy in the rehabilitation process of people diagnosed with spinal cord injury.

## Supplementary information

Supplemental Material. Figure 1.

Supplemental Material. Table 1.

Supplemental Material. Table 2.

## Data Availability

All data generated or analyzed during the current study are included in this article and its Supplementary Information files.
