# Supplementary material for: Health literacy of people with spinal cord injury: a systematic review
Source: Spinal Cord. 2023 Jun 30;61(8):409–14. doi: 10.1038/s41393-023-00903-4 (PMC10432272; doi:10.1038/s41393-023-00903-4)
Supplement: Supplementary file 1 — Supplemental Material. Figure 1. [file 41393_2023_903_MOESM1_ESM.docx]

Record identified from:

Pubmed (683)

Embase (520)

Cochrane (60)

Web of Science (135)

(n=1398)

**Identification**

Recors removed before screening:

Duplicate records removed (n=392)

Records screened

**(**n=1006)

Records removed by elegibility criteria (n= 995)

**Screening**

Reports sought for retrieval

(n=11)

Reports excluded:

-Qualitative studies (n=3)

- Not related to population of interest (n=1)

-Summary presented in congress (n=1)

- Article did not mention the instrument for assessing helth literacy (n=1)

Reports assessed for eligibility

(n= 11)

Studies included in review

(n=5)

**Included**
