# Supplementary material for: Health literacy of people with spinal cord injury: a systematic review
Source: Spinal Cord. 2023 Jun 30;61(8):409–14. doi: 10.1038/s41393-023-00903-4 (PMC10432272; doi:10.1038/s41393-023-00903-4)
Supplement: Supplementary file 2 — Supplemental Material. Table 1. [file 41393_2023_903_MOESM2_ESM.docx]

Supplementary Material Appendix 1

Table 1: Search Strategy

| PUBMED  (Capes Periodical Portal)  december 2021  1974 to december 2021  Search Strategy | 1 "health literacy" [MeSH Terms] OR ("health" [All Fields] AND "literacy" [All Fields]) OR "health literacy" [All Fields] OR ("literacies" [All Fields] OR "literacy" [MeSH Terms] OR "literacy" [All Fields] OR "literacy" [All Fields]) OR ("patient education handout" [Publication Type] OR "patient education as topic"[MeSH Terms] OR "patient education" [All Fields])  2 "health literacy" [All Fields] OR "literacies" [All Fields] OR "literacy" [MeSH Terms] OR "literacy" [All Fields] OR "literacy" [All Fields] OR "patient education as topic" [All Fields] OR "health education" [All Fields]  3 (("spinal cord" [MeSH Terms] OR ("spinal" [All Fields] AND "cord" [All Fields]) OR "spinal cord" [All Fields]) AND ("injure" [All Fields] OR "injured" [All Fields] OR "injures" [All Fields] OR "injuring" [All Fields])) OR ("spinal cord injuries" [MeSH Terms] OR ("spinal" [All Fields] AND "cord" [All Fields] AND "injuries" [All Fields]) OR "spinal cord injuries" [All Fields] OR ("spinal" [All Fields] AND "cord"[All Fields] AND "injury" [All Fields]) OR "spinal cord injury" [All Fields]) OR ("spinal cord" [MeSH Terms] OR ("spinal" [All Fields] AND "cord" [All Fields]) OR "spinal cord" [All Fields]) OR ("quadriplegia"[MeSH Terms] OR "quadriplegia" [All Fields] OR "quadriplegias"[All Fields]) OR ("paraplegia" [MeSH Terms] OR "paraplegia" [All Fields] OR "paraplegias"[All Fields]) OR ("paraplegia" [MeSH Terms] OR "paraplegia" [All Fields] OR ("paraplegia" [All Fields] AND "spinal" [All Fields] OR "paraplegia spinal"[All Fields]  4 1 or 2  5 4 and 3  Limit to 5 yr="1974 -2020"  Hits:683 |
| --- | --- |
| EMBASE  (Capes Periodical Portal)  december 2021  1974 to december 2021  Search Strategy | 1 'health literacy'/exp OR 'health literacy' OR 'literacy'/exp OR literacy OR 'patient education'/exp OR 'patient education' 541 artigos.  2 'health literacy'/exp OR 'health literacy' OR 'literacy'/exp OR literacy OR 'medical information'/exp OR 'medical information' OR 'health education'/exp OR 'health education'  3 'spinal cord injuries' OR 'spinal cord injury'/exp OR 'spinal cord injury' OR 'spinal cord'/exp OR 'spinal cord' OR 'quadriplegia'/exp OR quadriplegia OR 'paraplegia'/exp OR paraplegia OR 'paraplegia spinal'  4 1 or 2  5 4 and 3  Limit 5 to yr="1974 -2020"  Hits: 520 |
| Web of Science  (Capes Periodical Portal)  december 2021  1974 to december 2021  Search Strategy | 1**"**health literacy" (Todos os campos) or literacy [All Fields] or "patient education as topic" [All Fields] 2"health literacy" [All Fields] or literacy [All Fields] or "medical information" [All Fields] or "health education" [All Fields]  3'spinal cord injuries' [All Fields] or 'spinal cord injury' [All Fields] or 'spinal cord' [All Fields] or 'quadriplegia' [All Fields] or 'paraplegia' [All Fields]  or 'paraplegia spinal' [All Fields]  4 #1 OR #2  5 #4 AND #3  Limit 5 to yr="1974 -2020"  Hits: 135 |
| Cochrane  (Capes Periodical Portal)  december 2021  1974 to december 2021  Search Strategy | #1(“health literacy”): ti,ab,kw OR (literacy):ti,ab,kw OR (“patient education as topic”):ti,ab,kw"  #2("health literacy"): ti,ab,kw OR (literacy):ti,ab,kw OR ("health information"):ti,ab,kw OR ("health education"):ti,ab,kw  #3("spinal cord injuries"): ti,ab,kw OR ("spinal cord injury"):ti,ab,kw OR (spinal cord):ti,ab,kw OR (tetraplegia):ti,ab,kw OR (paraplegia):ti,ab,kw"  Hits: 60 |
