# Supplementary material for: Health literacy of people with spinal cord injury: a systematic review
Source: Spinal Cord. 2023 Jun 30;61(8):409–14. doi: 10.1038/s41393-023-00903-4 (PMC10432272; doi:10.1038/s41393-023-00903-4)
Supplement: Supplementary file 3 — Supplemental Material. Table 2. [file 41393_2023_903_MOESM3_ESM.docx]

| Checklist | Hahn et al., 2017 | Hogan et al., 2015 | Johnston et al., 2005 | Myaskovsky et al., 2011 | Sertkaya, et al.  2021 |
| --- | --- | --- | --- | --- | --- |
| Were the criteria for inclusion in the sample clearly defined? | Yes | Yes | Yes | Yes | No |
| Were the study subjects and the setting described in detail? | No | Yes | Yes | Yes | Yes |
| Was the esposure messured in a valid and reliable way? | Yes | Yes | Yes | Yes | Yes |
| Were objective, standard criteria used for measurement of the condition? | Yes | Yes | Yes | Yes | Yes |
| Were confounding factors identified? | Yes | Yes | Yes | Yes | Yes |
| Were strategies to deal with confounding factors stated? | Yes | Yes | Yes | Yes | Yes |
| Were the outcomes measured in a valid and reliable way? | Yes | Yes | No | Yes | Yes |
| Was Was appropriate statistical analysis used? | Yes | Yes | Yes | Yes | Yes |
| Overall Appraisal | Include | Include | Include | Include | Include |

Supplementary Table 2- JBI Checklist for Analytical Cross Sectional Studies
